# Supplementary material for: Assessing morphological variations in the seagrass genus Halodule (Cymodoceaceae) along the Brazilian coast through genetic analyses
Source: PeerJ. 2025 Mar 19;13:e19038. doi: 10.7717/peerj.19038 (PMC11929505; doi:10.7717/peerj.19038)
Supplement: Supplemental Information 1 [file peerj-13-19038-s001.docx]

Supplementary table 1. Sampling information.

| **Site** | **Coordenates** | | **Ecosystem** | **Samples (morphological)** | **Samples (molecular)** | | **GenBank accession No.** | | **Haplotype** | **Leaf tip** |
| --- | --- | --- | --- | --- | --- | --- | --- | --- | --- | --- |
|  | **Lat** | **Long** |  |  | **ITS** | **rbcL** | **ITS** | **rbcL** |  |  |
| Pedra do Sal (PS) | -2.81359722 | -41.72624722 | Beach | 10 | 5 | 5 | OR284886 | OR345359 | Hap_1 | 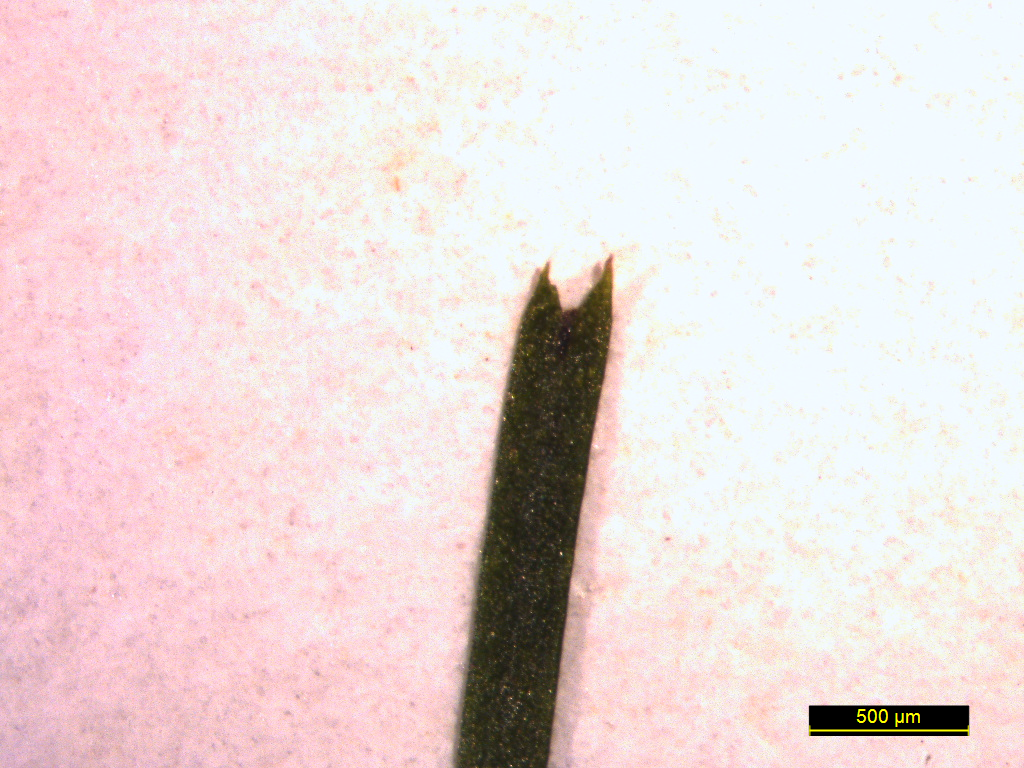 |
| Praia de Macapá (MAC) | -2.91486944 | -41.45075278 | Estuary | 10 | 5 | 4 | OR284887 | OR345360 | Hap_2 |  |
| Timonha-Ubatuba (SETU) | -2.96099444 | -41.32233611 | Estuary | 10 | 4 | 5 | OR284885 | OR345358 | Hap_1 |  |
| Praia de Manguinhos (PM) | -22.76953333 | -41.91093333 | Coastal lagoon | 10 | 10 | 10 | OR284889 | OR345362 | Hap_2 | 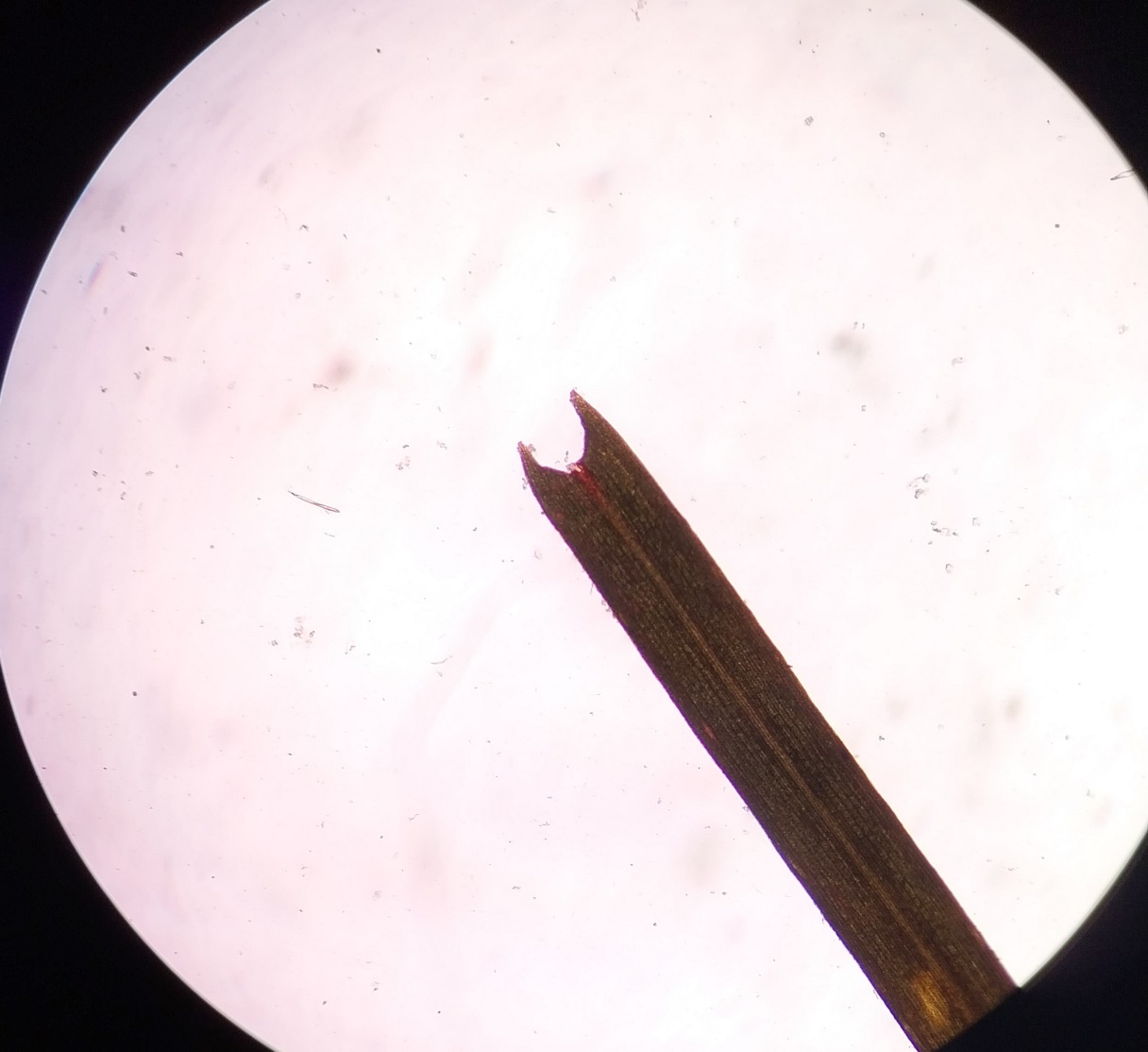 |
| Lagoa da Conceição (LC) | -27.59112778 | -48.43615556 | Beach | 10 | 6 | 6 | OR284888 | OR345361 | Hap_2 | 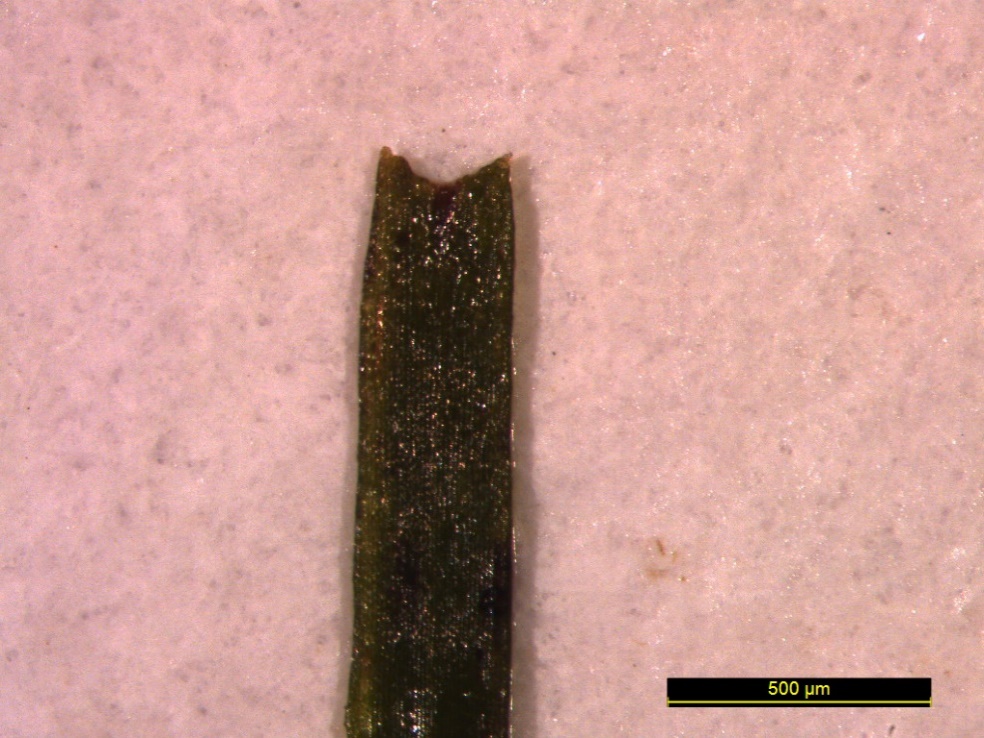 |
