# Supplementary material for: Assessing morphological variations in the seagrass genus Halodule (Cymodoceaceae) along the Brazilian coast through genetic analyses
Source: PeerJ. 2025 Mar 19;13:e19038. doi: 10.7717/peerj.19038 (PMC11929505; doi:10.7717/peerj.19038)
Supplement: Supplemental Information 2 [file peerj-13-19038-s002.docx]

Supplementary table 2. AMOVA results for the five populations and the groups formed by the northeast (PS, MAC and SETU) and south (PM and LC) regions.

|  | Among populations within groups | Within populations | Total |
| --- | --- | --- | --- |
| d.f. | 4 | 20 | 24 |
| Sum of squares | 2.000 | 10.000 | 12.000 |
| Variance components | 0.000 | 0.500 | 0.500 |
| Percentage of variation | 0.00 | 100.00 |  |
| F-statistics p-values | 1.000 | 1.000 |  |

|  | Among groups | Among populations within groups | Within populations | Total |
| --- | --- | --- | --- | --- |
| d.f. | 1 | 3 | 20 | 24 |
| Sum of squares | 0.500 | 1.500 | 10.000 | 12.000 |
| Variance components | 0.000 | 0.000 | 0.500 | 0.500 |
| Percentage of variation | 0.00 | 0.00 | 100.00 |  |
| F-statistics p-values | 1.000 | 1.000 | 1.000 |  |
