# Supplementary material for: Assessing morphological variations in the seagrass genus Halodule (Cymodoceaceae) along the Brazilian coast through genetic analyses
Source: PeerJ. 2025 Mar 19;13:e19038. doi: 10.7717/peerj.19038 (PMC11929505; doi:10.7717/peerj.19038)
Supplement: Supplemental Information 4 — Modifications in this study. [file peerj-13-19038-s004.docx]

CTAB Protocol

The leaves were macerated with liquid nitrogen in a mortar and pestle; the macerate was transferred to 2 ml microtubes with 700 μl of 2x CTAB buffer (1.4 M NaCl; 100 mM Tris-HCl (pH 8); 20 mM EDTA; 2% CTAB; 0.2% 2-mercaptoethanol) previously heated to 65°C, and then incubated in a water bath at 60°C for 60-120 min and homogenised every 10 min. We added 700 μl of chloroform:isoamyl alcohol (24:1) to the microtubes, mixed in a vortex mixer for 1 min, and centrifuged at 12,000 rpm for 10 min. The supernatant was transferred to a new 1.5 ml microtube and 600 μl of cold isopropanol was added. The tubes were inverted 50 to 60 times and left in the freezer (-20°C) overnight, we then centrifuged the solution at 12,000 rpm for 10 min and discarded the supernatant. The pellet was washed twice, the first time with 700 μl of 70% ethanol leaving it for 20 min and the second time with 500 μl of 100% ethanol, centrifuging at 12,000 rpm for 5 min and pouring off the supernatant during each wash. For elution, 30 μl of TE buffer and 2 μl of RNAse are added to the pellet and then stored in the freezer. To visualise the integrity of the extracted DNA, a 1% gel electrophoresis run was carried out and its quantification and purity were measured using a BioDrop spectrophotometer.
